# Bioactive Glucosinolate‐Rich Extract Promotes Growth in Broccoli Seedlings by Modulating Energy Allocation

**DOI:** 10.1111/ppl.70391

**Published:** 2025-07-12

**Authors:** Lorena Albaladejo‐Marico, Micaela Carvajal, Lucia Yepes‐Molina

**Affiliations:** ^1^ Aquaporins Group, Centro de Edafologia y Biologia Aplicada del Segura, CEBAS‐CSIC Murcia Spain

**Keywords:** *Brassica oleracea* L., glucosinolates, seed priming, transcriptomic

## Abstract

Bioactive extracts derived from plants are emerging as the most innovative and promising alternative to traditional stimulants/chemicals in the agricultural market, given their high availability and rich content of bioactive compounds. Previously, the group synthesized and characterized a Brassicacea extract rich in secondary metabolites such as glucosinolates and phenols, which demonstrated a biostimulant effect in broccoli ( *Brassica oleracea* L. var. *italica*) seedlings. Consequently, the main objective of this study was to investigate in detail the molecular mechanisms responsible for the stimulant capacity exhibited by the Brassicacea extract. For this aim, RNA sequencing was carried out to analyze gene expression in broccoli seedlings grown with the extract for 2 weeks, in combination with physiological measurements related to mineral composition, root transport, photosynthesis, and secondary metabolism. Treated seedlings exhibited an increase in macronutrients such as K, Ca, Mg, and S, along with a significant up‐regulation of aquaporin genes and an enhancement in relative water content (RWC), indicating a clear improvement in mineral and water homeostasis. Additionally, root structure was enhanced, correlating with the overexpression of genes associated with suberin synthesis. Moreover, a general activation of genes involved in energy production, including those of the Krebs cycle, was observed. The results revealed that the extract plays a key role in modulating plant metabolism by shifting resources away from secondary metabolism and redirecting them toward primary metabolism, ultimately promoting a higher growth rate.

## 1 Introduction

Secondary metabolites play a fundamental role in plant development, acting as defense molecules against abiotic and biotic stresses (Chowdhury 2022). Although their presence is not essential for plant growth, they are fundamental for the survival of plant species in different environmental conditions (Del Carmen Martínez‐Ballesta et al. 2013). In the Brassicaceae family, glucosinolates and phenolic compounds are the most abundant secondary metabolites. Both have generated great interest due to their bioactive properties and their applications in food and medicine (García‐Ibañez et al. 2022) and recently, in agriculture (Nicolas‐Espinosa et al. 2023).

In agriculture, phenols are gaining a prominent role because of their antioxidant capacity and potential use as biostimulants in different crops (Kisiriko et al. 2021). In the case of glucosinolates, although their human nutritional and plant defensive benefits are well known (Cartea and Velasco 2008), their potential use as biostimulants has remained largely unexplored. Recently, Albaladejo‐Marico et al. (2024) reported an increase in broccoli seedling development following the application of an extract rich in these metabolites, as well as after the application of pure glucosinolates such as glucoraphanin and glucobrassicin. This finding suggested that glucosinolates may have a significant role as growth stimulants in crops, opening new avenues for research and application in agriculture.

Plant‐derived biostimulants have been reported to enhance crop growth and resistance by modulating the metabolism of treated plants, altering the expression of key genes, and promoting the synthesis of protective and growth‐promoting compounds (Ben Mrid et al. 2021). By optimizing resource use efficiency, biostimulants have been reported to allow plants to redirect part of their energy toward growth, production, and defense without excessive expenditure of metabolic resources (Popko et al. 2018).

A key aspect in plant production is seed germination, a critical process that determines the final yield of crops. Increasing the germination rate and accelerating the transfer of crops to the field are priority objectives in the agricultural sector (Wozniak et al. 2020). In this respect, the technique of *seed biostimulation or priming*, which can improve the stress tolerance of seeds, has been shown to be a promising tool. A “priming memory” is established during priming, initiated by modifications in transcription factors and epigenetic mechanisms. Such changes lead to alterations in transcript levels, which subsequently influence metabolite synthesis. This memory can subsequently be activated when seeds are confronted with stress conditions during germination (Chen and Arora 2013). This technique not only increases seed resistance in the early stages of development but can also positively influence their secondary metabolism, maximizing crop yield and resilience (Ali et al. 2019; Chakraborti et al. 2022).

The present study investigated the effects of a plant extract rich in glucosinolates and phenolics on the gene expression, metabolism, and physiology of broccoli ( *Brassica oleracea* L. var. *italica*) seedlings. This study included the analysis of various physiological parameters, such as photosynthesis, water and mineral transport, root structure, and glucosinolate uptake. Additionally, a transcriptomic analysis was performed to elucidate the mechanisms by which the extract stimulates seedling growth and metabolic adaptation. These findings provide relevant information for the development of plant biostimulants in the agricultural field to face specific problems.

## 2 Methods

### 2.1 Seed Growth and Treatment In Vitro

Broccoli seeds were sterilized with sodium hypochlorite:water (1:1) and placed in Petri dishes (15 × 100 mm) with 20 mL of agar‐water media (0.8% PhytoAgar). A broccoli extract, previously characterized in Albaladejo‐Marico et al. (2024), was applied to agar‐water media at a concentration of 1:40. Ten seeds were placed in each plate and grown in a cultivation chamber for 13 days. The conditions included a 16/8‐h light/dark cycle, with temperatures of 25°C and 20°C, respectively. The relative humidity was set at 60% during the day and 80% during the night, and the photosynthetically active radiation (PAR) was maintained at 400 μmol m^−2^ s^−1^ provided by LEDs (Pacific LED, WT 470C, LED8OS/840 PSD WB L1600 lights, Philips). After 13 days of growth, the aerial parts of the seedlings were separated from the roots for subsequent assays.

### 2.2 Physiological Measurements

The relative water content (RWC) was calculated by the following formula: RWC = [(FW—DW)/(TW—DW)] × 100, where FW is the fresh weight, DW is the dry weight, and TW is the turgid weight (Smart and Bingham 1974). The osmotic potential (Ψs) was quantified using a freezing‐point depression osmometer (Digital Osmometer, Roebling) at 25°C ± 1°C (Navarro et al. 2003). Photosynthetic capacity was measured in the complete plantlet using a portable photosynthesis system (LI‐8400, Li‐Cor Inc.) equipped with an integrated fluorescence chamber head 6800‐01A Leaf Chamber. Measurements and data analysis were performed by the Plant Biotechnology Service from ACTI of University of Murcia.

### 2.3 Mineral Content Analysis

Aerial parts and roots of broccoli seedlings were lyophilized to determine the mineral content at the Ionomics Laboratory (CEBAS‐CSIC, Murcia, Spain) as described in Nicolas‐Espinosa et al. (2024), using inductively coupled plasma‐optical emission spectrometry (ICP‐OES).

### 2.4 RNA Isolation, Library Assembly and Transcriptome Analysis

Both aerial and root parts were grinded in liquid nitrogen. Total RNA was extracted using the NucleoSpin RNA Plant and Fungi (Macherey‐Nagel), following the manufacturer's recommended protocol. The integrity of the RNA obtained was measured using an Agilent Bioanalyzer 2100 system (Agilent Technologies) with a 2100 expert Eukaryote total RNA Nano Chip before library construction. The cDNA libraries were generated using the Illumina Stranded mRNA Prep, Ligation kit (Illumina) according to the manufacturer's instructions. The libraries were quantified using the dsDNA High Sensitivity Assay on a Qubit 4 fluorometer (Thermo Fisher Scientific). The quality of the libraries was validated by assessing the fragment length with an Agilent 2100 using the D1000 High Sensitivity assay (Agilent Technologies). Library preparation and transcriptome sequencing were carried out at the Molecular Biology Services (ACTI, University of Murcia, Spain) on a NextSeq 1000/2000 system and P3 flow cell chemistry, generating 75 bp paired end reads with a minimum of 25 million reads per sample.

### 2.5 RNAseq Data Analysis

Preprocessing of raw Illumina RNASeq reads was performed with *fastp* (v0.20.0) (Chen et al. 2018), aimed at filtering out low‐quality sequences. The quality of the reads, both pre‐ and post‐fastp processing, was assessed using *FastQC* (v0.11.9) (http://bioinformatics.bbsrc.ac.uk/projects/fastqc). Clean reads were aligned to the *Broccoli reference genome* (Braol HDEM V1.0, downloaded from http://brassicadb.cn/#/ (Chen, Wang, et al. 2022)) using *HISAT2* (v4.8.2) (Kim et al. 2015). The resulting SAM files were converted to BAM format and sorted using *Samtools* (v0.1.19) with default parameters (Li 2009). *FeatureCounts* (v2.0.1) from the subread package was employed to quantify read counts for each sample (Liao et al. 2014). Normalized gene expression levels were obtained using *variance stabilizing transformation*, and differentially expressed genes (DEGs) were identified using a *q*‐value ≤ 0.05 and log2 (fold change) thresholds of < −1 or > 1, as calculated by *DESeq2* (Love et al. 2014).

Principal component analysis (PCA) was applied to the rlog‐transformed count data for all genes, and results were visualized with the *plotPCA* function from *DESeq2*. Additionally, heatmaps were created using the *ComplexHeatmap* R package (v1.20.0).

A KEGG analysis (https://www.kegg.jp/kegg/pathway.html) was performed to select the metabolic pathways of greatest interest (Figure S1A,B). The gene lists obtained from the KEGG analysis were expanded through additional literature review and searches in the *BnlR* database (https://yanglab.hzau.edu.cn/BnIR). Subsequently, all selected gene codes were annotated using the *TAIR* (https://www.arabidopsis.org/) and *Ensembl Plants* (https://plants.ensembl.org/index.html) databases.

For GO terms, the *Brassica napus* multi‐omics information resources (https://yanglab.hzau.edu.cn/BnIR) were used, with the var_italica_HDEM genome as a reference. Those categories related to biological processes (BP), with the most significant *p* value, were selected.

### 2.6 Kinetic Essay

In the kinetics test, broccoli seeds were germinated under the same circumstances as previously described. Additionally, seedlings were collected on days 1, 3, 7, and 10, and secondary metabolites were extracted from the aerial part, the root of fresh seedlings, and the agar on which they were grown. The extraction and analysis method used to detect glucosinolates in seedlings and agar was previously described by Albaladejo‐Marico et al. (2024).

### 2.7 Detection of Suberin in Roots

For the detection of suberin in secondary roots of broccoli seedlings, the protocol previously described by Cohen et al. (2020) was followed. The fluorophore FY 088 (0.01% w/v in lactic acid) was used. Cell walls were then washed and stained with propidium iodide (PI) (0.01% w/v in ddH_2_O). GFP and TRITC filters were used to view suberin deposits. To observe the root structure, cross sections of 10 μm made with the cryostat were excited with UV light (405 nm). All samples were observed under the STELLARIS 8 confocal microscope of the Microscopy and Image Analysis Service from SACE of the University of Murcia.

### 2.8 Statistical Analysis

Statistical analyses and data presentation were conducted using Origin (Pro), Version 2021 (OriginLab Corporation). Tests were preceded by a normality test and a Grubbs test to identify potential outliers. All the data were subjected to a *t*‐test. Significance levels are denoted as follows: * for *p* < 0.05, ** for *p* < 0.01, *** for *p* < 0.001, and n.s. for no significant differences. All presented values represent the means ± SE. Heatmaps were generated using R 4.3.1 software (R Core Team 2021), the gplots (Warnes et al. 2016) and pheatmap packages (Kolde 2012) were used, and FactoMineR (Lê et al. 2008) and factoextra (Kassambara and Mundt 2020) were used to generate Principal Component Analysis (PCA).

## 3 Results

### 3.1 Analysis of RNAseq Samples

**FIGURE 1:**
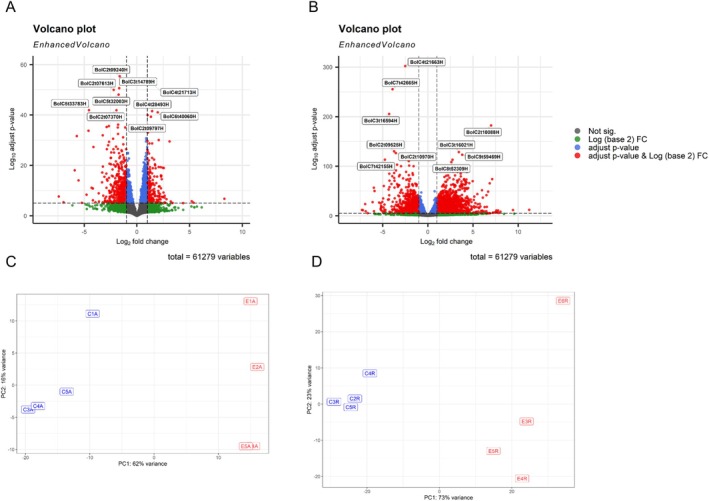
Differentially expressed genes (DEGs) from control and extract‐treated broccoli seedlings. DEGs in both (A) aerial and (B) root part were represented using volcano plots. The *x*‐axis represents the fold‐change (Log_2_), while the y‐axis represents the significance level, with *p* values indicating the extent of changes in gene expression. Each point on the graph represents an individual gene, blue indicates genes with a *p* value < 0.05, green indicates a |fold change| > 1, red indicates a *p* value < 0.05 and a |fold change| > 1 and gray those genes that showed no significant difference in expression. Principal component analysis (PCA) plot for the RNA‐seq data of the biological replicates of the control and treated seedlings, in (C) the aerial part and (D) root.

The gene expression of *Brassica oleracea* L. var. *italica* (broccoli) seedlings was analyzed under control conditions and after plant extract treatment (Table S1). A total of 61,279 genes were analyzed using an adjusted *p* value < 0.05 and a |fold change| > 1. In the aerial part, 662 genes were up‐regulated and 1292 down‐regulated (Figure 1A), while a greater number of changes were observed at the root level; 1385 genes were down‐regulated and 2731 up‐regulated (Figure 1B). In addition, variability between treatments and replicates was assessed by principal component analysis (PCA). This analysis showed a clustering of the control replicates on the one hand and those treated with the extract on the other hand, both in the aerial part (Figure 1C) and in the root (Figure 1D).

### 3.2 Analysis of the Most Affected Metabolic Pathways

**FIGURE 2:**
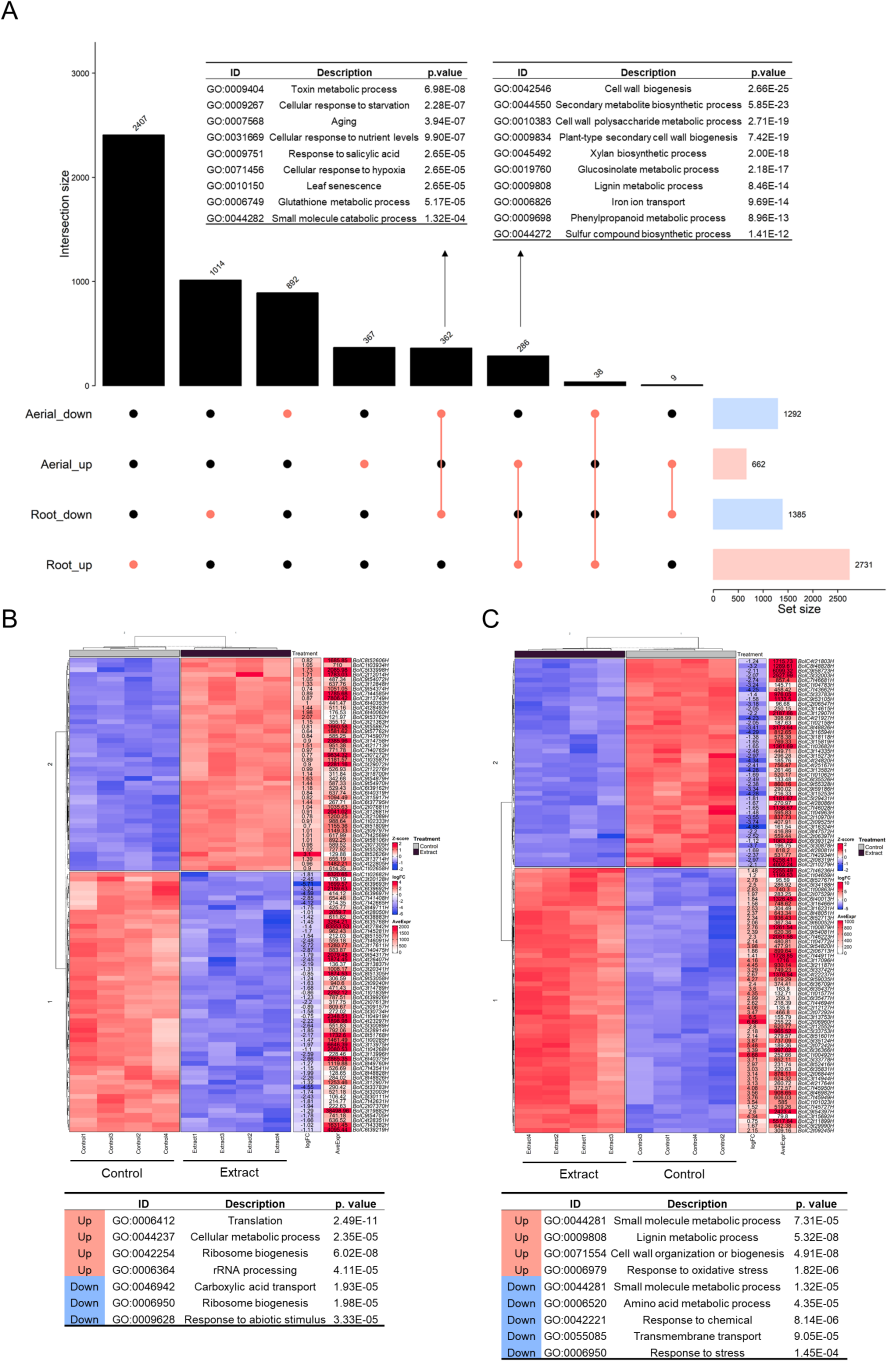
Functional analysis of altered metabolic pathways. (A) Interaction plot, representing the differentially expressed genes in the aerial part and root of broccoli seedlings treated with extract, compared to the control. In addition to the GO terms of biological processes (BP) of the most significant genes that are down‐regulated and up‐regulated in both parts. (B) Heatmap of top 100 genes with most significant *p* value of the aerial part and associated GO terms (BP). (C) Heatmap of top 100 genes with most significant root *p* value and associated GO terms (BP). Blue represents genes with low expression and red represents genes with high expression (*Z*‐score), fold change (logFC), mean expression (AveExpr) and gene ID are shown.

An upset plot was carried out (Figure 2A), in which the differentially expressed genes in all conditions, control and extract‐treated broccoli seedlings, are represented. A total of 362 were down‐regulated, both in the aerial part and in the root, and 9 categories were identified in GO terms related to biological processes (BP), including toxin metabolic process (GO:0009404), cellular response to starvation (GO:0009267) and aging (GO:0007568). On the other hand, 286 genes were up‐regulated in both parts, identifying 10 categories in the GO terms BP. Among the most prominent categories are cell wall biogenesis (GO:0042546), secondary metabolite biosynthetic process (GO:0044550) and cell wall polysaccharide metabolic process (GO:0010383).

The 100 most significant *p* value genes from the aerial part (Figure 2B) and root (Figure 2C) were analyzed, and the most significant GO (BP) terms related to these genes were identified. In the aerial part, the biological processes with the highest number of up‐regulated genes included those related to translation (GO:0006412) and cellular metabolic processes (GO:0044237), while genes associated with carboxylic acid transport (GO:0046942) and stress response (GO:0006950) were found to be down‐regulated. In the root, the most notable up‐regulated processes involved lignin metabolic process and cell wall formation (GO:0009808, GO:0071554), whereas genes related to amino acid metabolic processes and response to chemicals were down‐regulated (GO:0006520 and GO:0042221).

### 3.3 Inhibition of Photosynthetic Activity and Gas Exchange in Treated Seedlings

**FIGURE 3:**
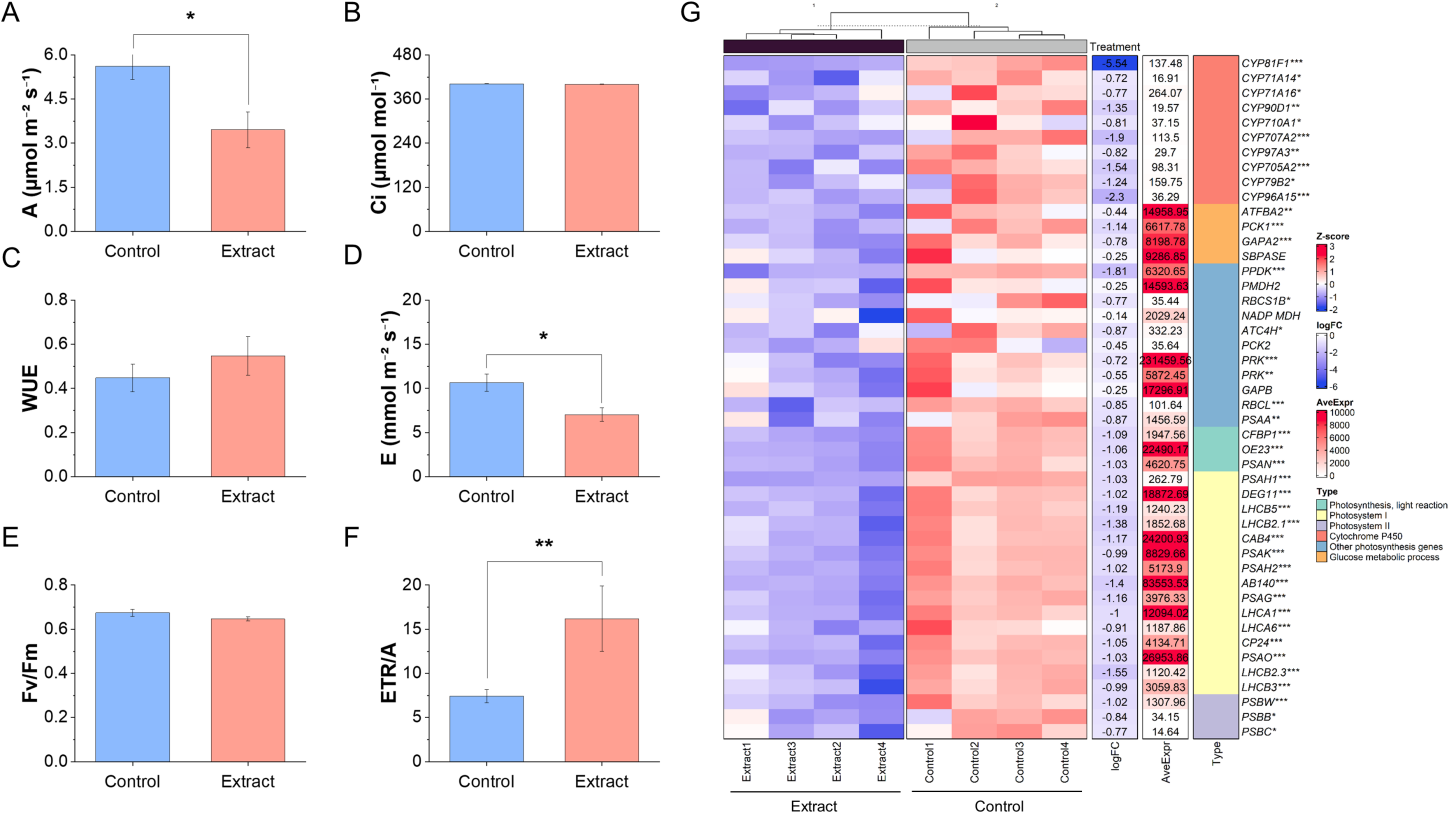
Analysis of main photosynthetic parameters and their related genes. (A) Net photosynthetic rate (A, μmol m^−2^ s^−1^), (B) calculated substomatal CO_2_ concentration (Ci, (A, μmol mol^−1^)), (C) water use efficiency (WUE), (D) evaporation (E, mmol m^−2^ s^−1^), (E) maximum quantum efficiency of photosystem II (Fv/Fm), and (F) electron transport rate (ETR/A). Each bar represents the mean ± SE (*n* = 10). (G) Heatmap of photosynthesis‐related genes in extract‐treated and control seedlings. Blue represents genes with low expression and red represents genes with high expression (*Z*‐score); fold change (logFC), mean expression (AveExpr) and gene name are shown. Significant differences between extract‐treated and control plantlets were measured by *t*‐tests. *p < 0.05, **p < 0.01, ***p < 0.001.

Different photosynthetic parameters were measured in the extract‐treated seedlings and the controls. Treated seedlings showed a lower net photosynthetic rate (A) and evaporation (E) (Figure 3A,D), as well as a higher electron transport rate ETR/A (Figure 3F). Additionally, a complete inhibition of gene expression of photosynthesis‐related genes was observed (Figure 3G), such as cytochrome P450 subunits and the small and large subunit of the rubisco enzyme (*RBCS1B* and *RBCL*).

### 3.4 Increase in Energy Production

**FIGURE 4:**
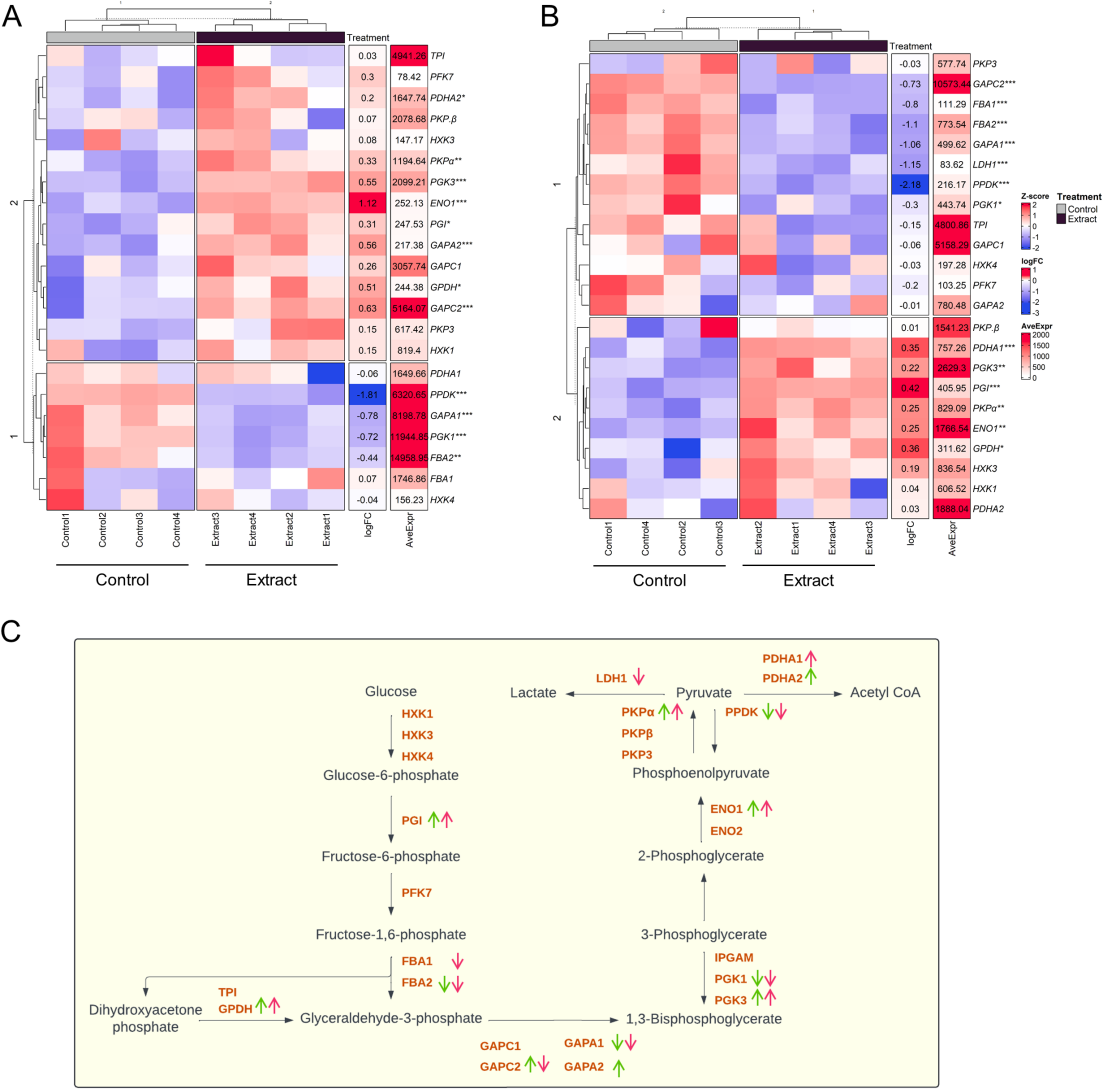
Analysis of glycolysis‐related gene expression in extract‐treated and control seedlings. Heatmap of glycolysis‐related genes in (A) the aerial part and (B) root. Blue represents genes with low expression, red represents genes with high expression (*Z*‐score), fold change (logFC), mean expression (AveExpr), and gene name are shown. Significant differences between extract‐treated and control plants were measured by *t*‐tests. *p < 0.05, **p < 0.01, ***p < 0.001. (C) Schematic representation of the glycolytic pathway, indicating changes in gene expression (up‐ or down‐regulation) in the aerial part (green) and root (pink).

Most of the main glycolysis genes (*PGI*, *ENO1*, *PKPα*, *PDHA1‐2*), in both the aerial and root parts (Figure 4A,B) were up‐regulated; however, the decreased expression of the *FBA* gene, which is involved in the conversion of fructose‐1,6‐bisphosphate to glyceraldehyde‐3‐phosphate, is notable. This activation pattern indicates an intensification of glycolytic flux, leading to the formation of pyruvate, which is subsequently converted to acetyl‐CoA (Figure 4C), a key intermediate for entry into the Krebs cycle.

**FIGURE 5:**
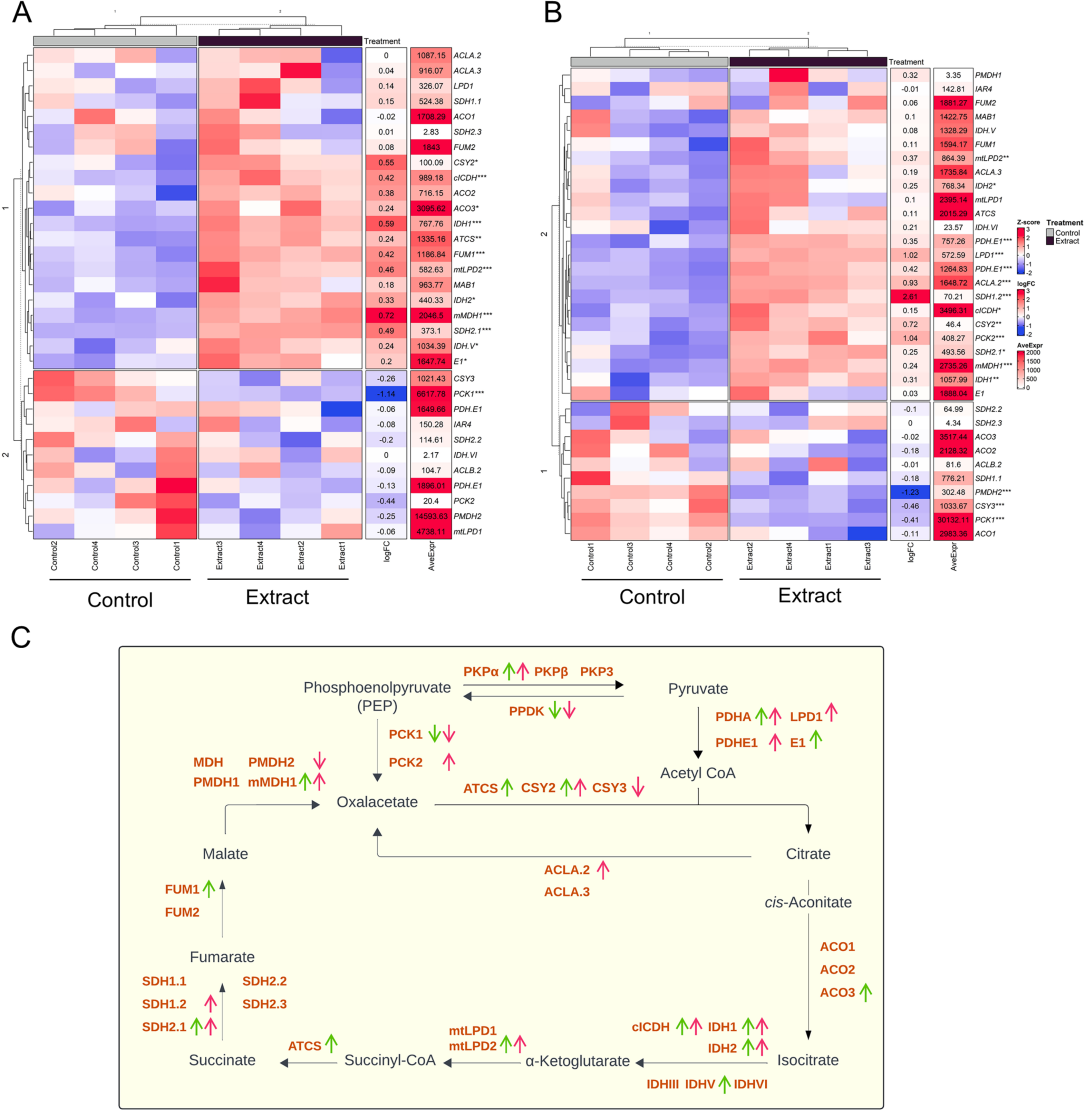
Analysis of Krebs cycle‐related gene expression in extract‐treated and control seedlings. Heatmap of Krebs cycle‐related gene expression in (A) the aerial part and (B) root. Blue represents genes with low expression and red represents genes with high expression (*Z*‐score); fold change (logFC), mean expression (AveExpr) and gene name are shown. Significant differences between extract‐treated and control plants were measured by *t*‐tests. *p < 0.05, **p < 0.01, ***p < 0.001. (C) Schematic representation of the Krebs cycle, indicating changes in gene expression (up‐ or down‐regulation) in the aerial part (green) and root (pink).

In addition, genes related to the Krebs cycle showed an up‐regulated expression pattern in both parts of the plant (Figure 5C), indicating increased formation of intermediates and energy generation. Genes related to the production of Krebs cycle intermediates, such as *PDHA*, *CSY*, *ACO*, *IDH*, *mtLPD*, *ATCS*, *SDH*, and *FUM*, were significantly up‐regulated (Figure 5A,B).

### 3.5 Enhanced Water Homeostasis and Mineral Uptake

**FIGURE 6:**
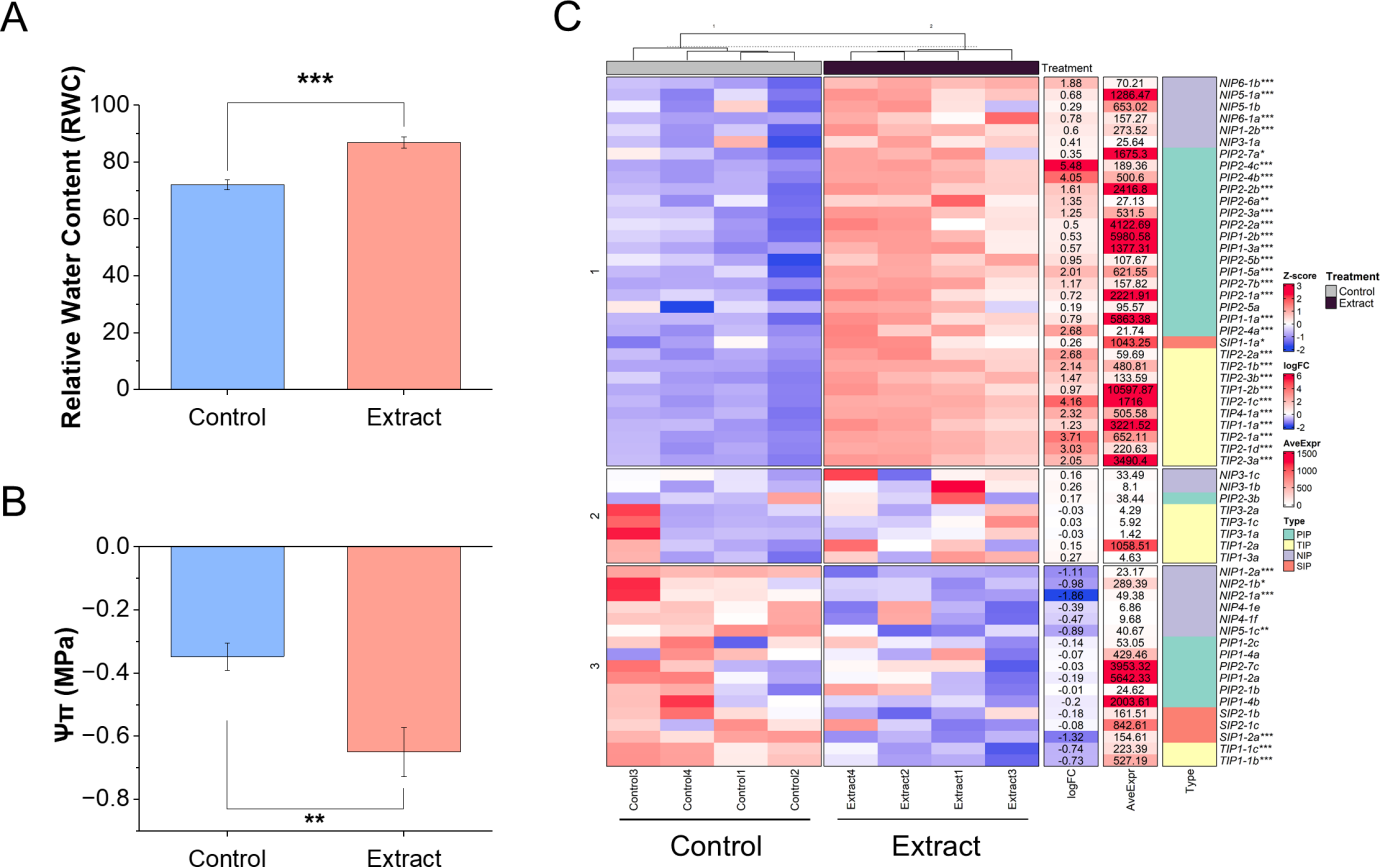
Water dynamics and aquaporin expression. (A) Relative water content (RWC) of the aerial part (*n* = 12), (B) osmotic potential (Ψs, MPa) (*n* = 12). Each bar represents the mean ± SE. (C) Heat map of aquaporins gene expression in extract‐treated and control seedlings in the root. Blue represents genes with low expression and red represents genes with high expression (*Z*‐score); fold change (logFC), mean expression (AveExpr), and gene name are shown. Significant differences between extract‐treated and control seedlings were measured by *t*‐tests. *p < 0.05, **p < 0.01, ***p < 0.001.

An increase in relative water content (RWC) (Figure 6A) along with a decrease in osmotic potential (Ψ_s_) (Figure 6B) was observed. Additionally, Figure 6C shows a heatmap revealing alterations in the expression of aquaporin genes in roots, with significant overexpression of most of them. Smaller expression changes were observed in the aerial part (Figure S2).

**FIGURE 7:**
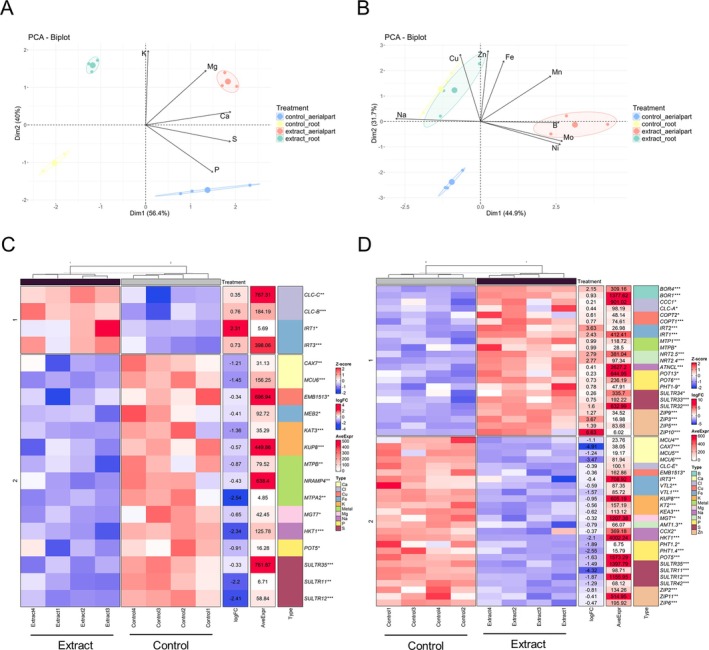
Analysis of mineral nutrients in the aerial part and root of broccoli seedlings treated with extract and control. Principal component analysis (PCA) of (A) macronutrients and (B) micronutrients with arrows indicating the loadings of each nutrient; 95% confidence ellipses were plotted for each treatment, each small circle representing data from an individual sample, while the large circle represents the centroid of the mean values. (C) Heatmap of mineral transporter gene expression in extract‐treated and control seedlings in the aerial part. (D) Heatmap of mineral transporter gene expression in extract‐treated and control seedlings in the root. Blue represents genes with low expression and red represents genes with high expression (*Z*‐score); fold change (logFC), mean expression (AveExpr) and gene name are shown. Significant differences between extract‐treated and control plants were measured by *t*‐tests. *p < 0.05, **p < 0.01, ***p < 0.001.

As shown by the multivariate PCA analysis, broccoli seedlings treated with the extract showed an alteration in the mineral profile, both macronutrients (Figure 7A) and micronutrients (Figure 7B). A significant increase in the concentration of macronutrients such as K, Ca, and Mg was observed both in the aerial part and the root, while S only increased in the root. However, P decreased in both parts (Figure S3A–E). As for micronutrients, high concentrations of Cu, Fe, Mn, Mo, Ni, and Zn were found in the aerial part, while B and Cu decreased in the root. In addition, treated seedlings showed a lower Na concentration in both aerial and root parts, thus decreasing the Na/K ratio drastically (Figure S3F–N).

Gene expression of different mineral transporters was analyzed (Figure S4), with only the significant ones shown in Figure 7C,D. In the aerial part, most of the significantly different transporters compared to the control were down‐regulated, except for some Cl (*CLC‐C*, *CLC‐B*) and Fe (*IRT1*, *IRT3*) transporters (Figure 7C). As for the significant genes in the root (Figure 7D), more heterogeneity was found, with some transporters up‐regulated and others down‐regulated, belonging to the same P, S, Zn, Na, Fe, and Cu group. Boron transporters (*BOR4* and *BOR1*) were found to be up‐regulated in roots, together with the metal transporters *MTP1* and *MTPB*. On the other hand, several Ca and K transporters were found to be down‐regulated.

### 3.6 Alteration of Secondary Metabolism

#### 3.6.1 Gene Expression

**FIGURE 8:**
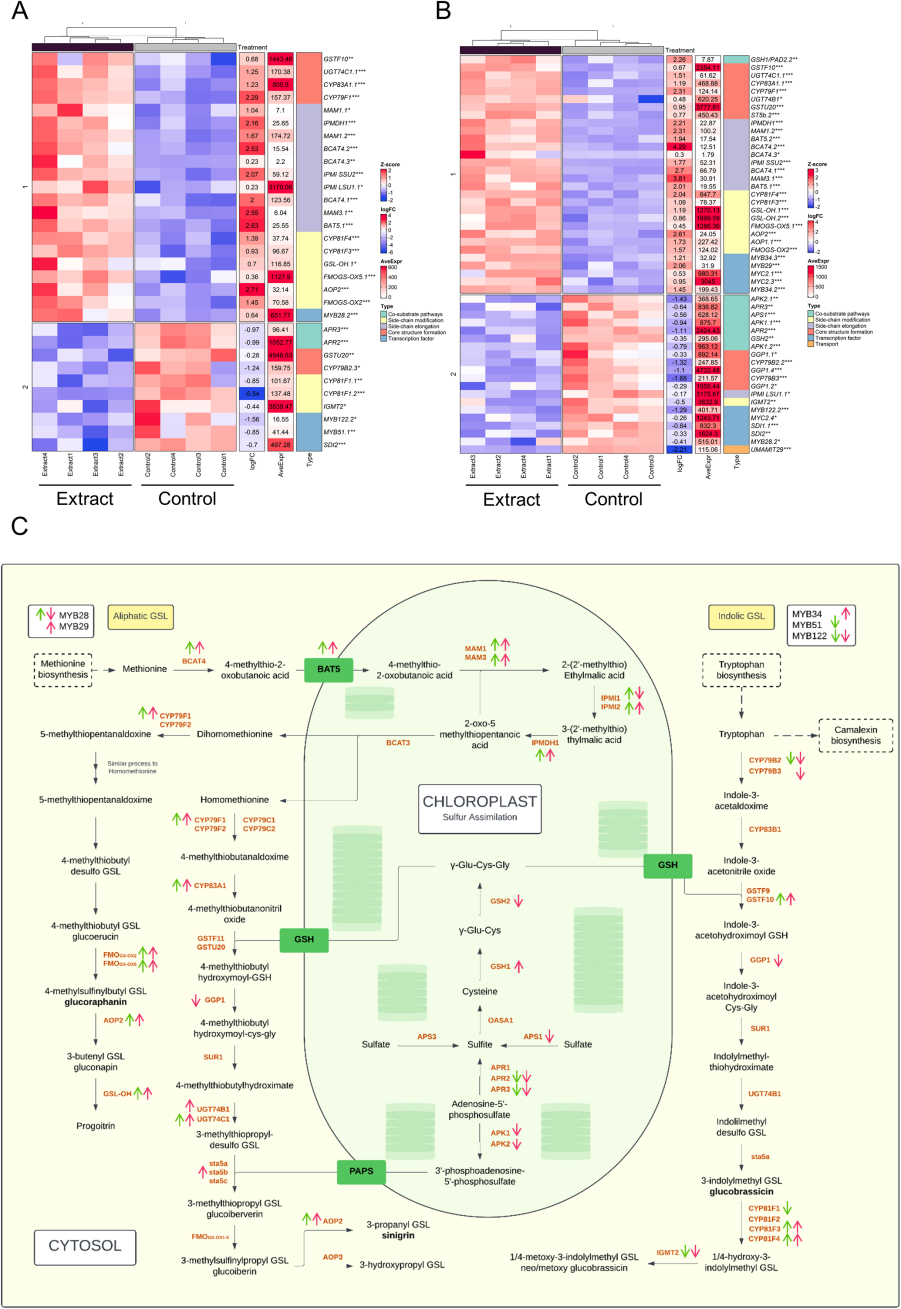
Analysis of glucosinolate synthesis and regulation‐related gene expression in extract‐treated and control seedlings. Heatmap of gene expression related to glucosinolate in (A) the aerial part and (B) root. Blue represents genes with low expression and red represents genes with high expression (*Z*‐score); fold change (logFC), mean expression (AveExpr) and gene name are shown. Significant differences between extract treated and control plants were measured by *t*‐tests. *p < 0.05, **p < 0.01, ***p < 0.001. (C) Schematic representation of glucosinolate synthesis, indicating changes in gene expression (up‐ or down‐regulation) in the aerial part (green) and root (pink).

Genes related to glucosinolate synthesis were analyzed (Figure S5A,B), and most of them were significantly altered in the aerial part (Figure 8A) and root (Figure 8B). Genes related to sulfur assimilation, such as *GSH2*, *APR2*, *APR3*, *APK1*, and *APK2*, were significantly down‐regulated, while genes involved in the synthesis of aliphatic glucosinolates were up‐regulated. Regarding indole glucosinolates, less significant differences were found, with some genes being up‐regulated and others down‐regulated.

Fewer changes and greater variability were observed in genes related to the synthesis of phenolic compounds, both in the aerial part and in the root (Figure S5C,D). A similar pattern was observed when analyzing genes related to the synthesis and regulation of gibberellins and zeatin (Figure S6C,D). In the case of auxins, genes involved in their synthesis showed an activation response (Figure S6A,B), especially in the root.

#### 3.6.2 Kinetic Essay

**FIGURE 9:**
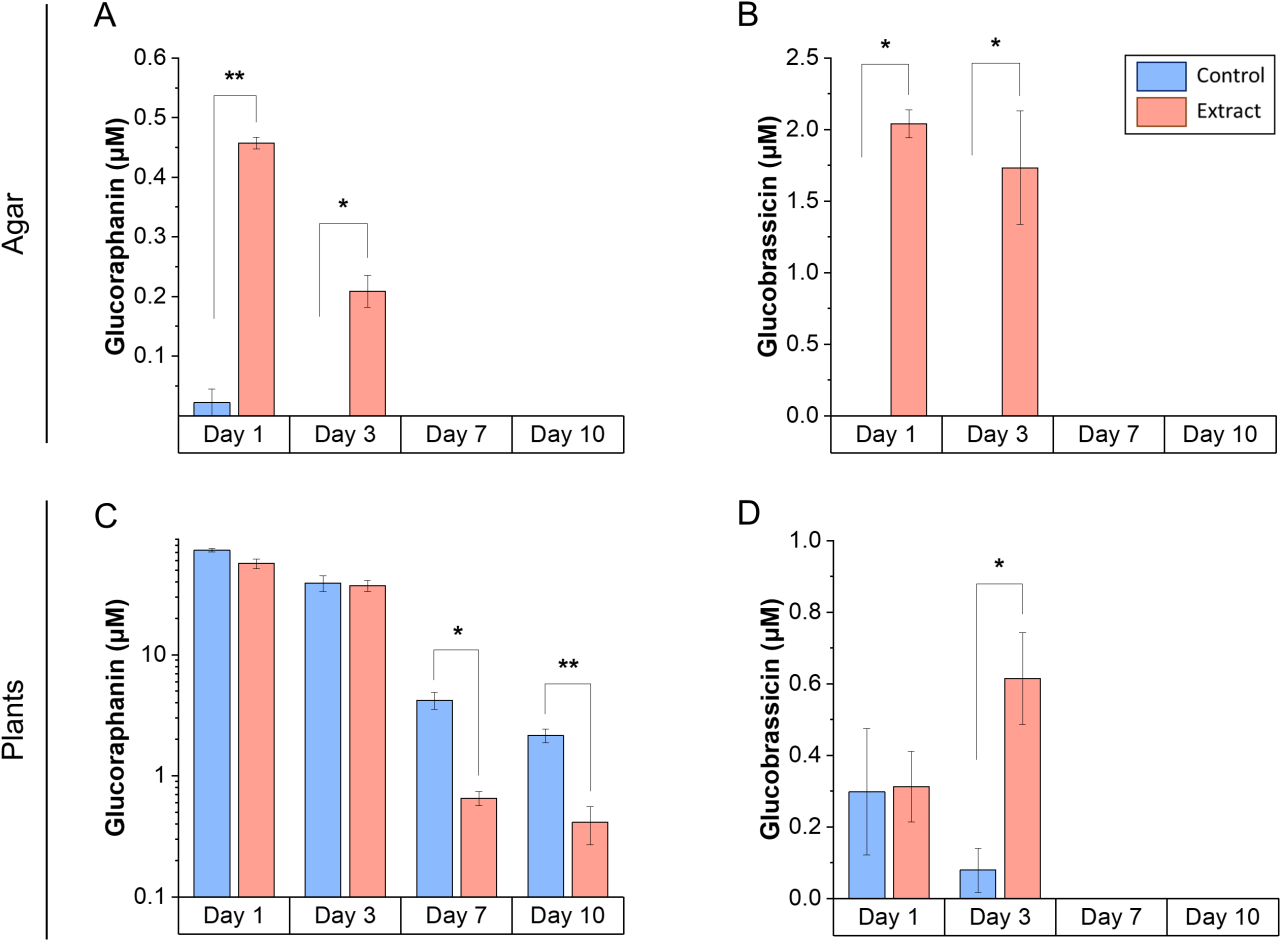
Kinetic assay of glucosinolate concentration. (A) Glucoraphanin and (B) glucobrassicin present in control agar and with added extract, after growth of broccoli seedlings on days 1, 3, 7, and 10. (C) Glucoraphanin and (D) glucobrassicin present in seedlings grown on control agar and with added extract on days 1, 3, 7, and 10. Each bar represents the mean ± SE (*n* = 3). Significant differences between control and extract treated agar/plants were measured by *t*‐tests. *p < 0.05, **p < 0.01, ***p < 0.001.

Glucosinolate levels were analyzed both in the agar of the Petri dishes and the seedlings under two experimental conditions: seedlings grown in agar with (red bar, Figure 9) and without plant extract (blue bar, Figure 9). Measurements were performed on days 1, 3, 7, and 10 to evaluate changes in concentrations over time.

Glucoraphanin disappeared from the agar between days 3 and 7 (Figure 9A), while its concentration in broccoli seedlings decreased significantly from day 7 onward (Figure 9C). Glucobrassicin also disappeared from the agar on day 7 (Figure 9B), but its concentration in the seedlings increased on day 3 and disappeared from day 7 onward (Figure 9D).

### 3.7 Improvement of Cell Organization and Root Length

**FIGURE 10:**
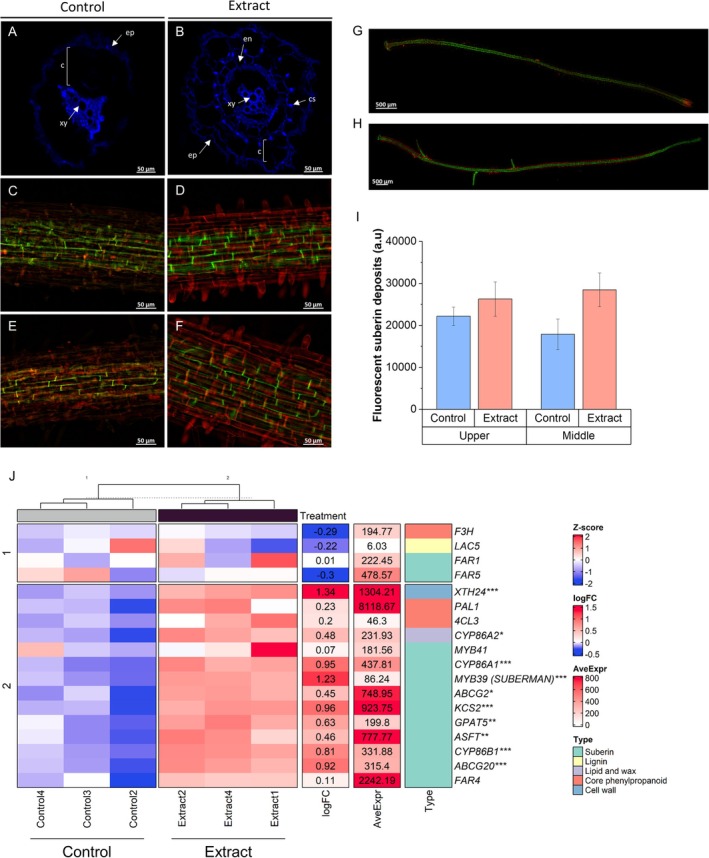
Extract‐treated seedlings showed greater root development. Autofluorescence when excited with UV light in cross‐section of a secondary root of (A) control and (B) extract‐treated seedlings. Arrows indicate different parts of the root: Epidermis (ep), cortex (c), xylem (xy), endodermis (en), and Casparian strip (cs). Observation by confocal microscopy of suberin (green) and cortex wall (red) deposits by staining with fluorol yellow (FY) and propidium iodide (PI), respectively; in the (C) upper part, (E) middle and (G) the hole root of the control, as well as in the (D) upper part, (F) middle, and (H) the hole root of the treated seedlings. (I) Measurement of fluorescence intensity of suberin deposits, each bar represents the mean ± SE of three biological replicates and three technical replicates. (J) Heatmap of the expression of genes related to root structure development in extract‐treated and control seedlings. Blue represents genes with low expression and red represents genes with high expression (*Z*‐score); fold change (logFC), mean expression (AveExpr) and gene name are shown. Significant differences between extract treated and control plants were measured by *t*‐tests. *p < 0.05, **p < 0.01, ***p < 0.001.

Root structure development was studied in extract‐treated seedlings and controls. Roots of extract‐treated seedlings (Figure 10B) showed more accelerated development than controls (Figure 10A) when autofluorescence of cross‐sections exposed to UV light (365 nm) was measured. Treated seedlings showed increased development of xylem, endodermis, and Casparian strip. In addition, suberisation of the endodermis was measured at the upper (Figure 10C,D) and middle (Figure 10E,F). Although no significant changes in the fluorescence intensity of suberin deposits were observed (Figure 10I), a more developed and organized structure was observed in the treated seedlings (Figure 10H) compared to the control (Figure 10G). In addition, a significant increase in the expression of genes related to suberin synthesis and deposition was observed, such as *MYB39* (*SUBERMAN*), *CYP86A1*, *CYP86B1*, *ABCG20*, and *XTH24* (Figure 10J).

## 4 Discussion

The present results build upon the findings reported by Albaladejo‐Marico et al. (2024), providing a molecular and physiological basis for the previously observed long‐term biostimulant effects. While the earlier study demonstrated that a short early treatment with the extract enhanced growth and metabolic activity into adulthood, the current data reveal the underlying mechanisms responsible for these sustained effects. Specifically, the transcriptomic and physiological analyses presented here show that the extract induces early molecular reprogramming, which likely contributes to the lasting developmental acceleration observed previously. These results not only validate the earlier phenotypic observations but also offer mechanistic insight into how early‐stage molecular reprogramming can drive long‐term improvements in plant performance.

The application of this extract in the previous work not only enhanced the development of treated seeds but also induced metabolic changes that persisted into adulthood. These plants maintained accelerated development despite receiving treatment only during the first 10 days (germination and seedling stages). To gain deeper insights into the effects of the glucosinolate and phenolics extract, the present study assessed various physiological parameters related to water and nutrient transport, alongside gene expression profiling via RNA sequencing, and identified the most significantly altered metabolic pathways, thus providing a comprehensive view of the extract's impact during the early seedling stage (10 days post‐germination).

A significant alteration in gene expression was observed in the aerial parts of treated seedlings (Figure 1A), reflecting a strategic reallocation of energy resources from photosynthesis to energy production and growth processes. This shift was evidenced by the down‐regulation of photosynthesis‐related genes, including *RBCS1B*, *RBCL*, and cytochrome P450 subunits, along with reductions in net photosynthetic rate (A) and evaporation (E) (Figure 3). The composition of the applied plant extract, rich in carbon sources such as organic acids, glucids, and amino acids (Albaladejo‐Marico et al. 2024), may contribute to this shift by providing alternative energy substrates, potentially inhibiting photosynthesis via feedback regulatory mechanisms (Paul and Pellny 2003). Concurrently, the up‐regulation of glycolysis‐related genes (*PDHA, PKP*) and Krebs cycle genes (*ATCS*, *CSY2*, *ACO3*, *ACLA2*, *ACO3*, *IDH1‐2*, *cICDH*, *ATCS*, *SDH2.1‐1.2 FUM1*, *mMDH1*) (Figures 4 and 5) indicates intensified metabolic flux, driving increased production of intermediates like citrate and succinate (Albaladejo‐Marico et al. 2024), which are critical for energy generation and biosynthesis of amino acids and secondary metabolites. This metabolic reprogramming likely underlies the rapid root development, enhanced nutrient transport, and improved organogenesis observed in treated seedlings, thereby supporting their growth and adaptation (Häusler et al. 2014).

The highest number of altered genes was found in the roots of the treated seedlings (Figure 1B). Structural enhancements observed in treated seedlings (Figure 10) were strongly supported by molecular changes (Figure 2), including accelerated root development evidenced through autofluorescence imaging. This revealed improved xylem and endodermis formation, as well as enhanced Casparian strip organization, which is critical for controlling nutrient and water uptake (Chapman et al. 2012). These structural improvements were further underpinned by the upregulation of genes involved in suberin biosynthesis and deposition, such as *MYB39* (SUBERMAN), *CYP86A1*, *CYP86B1*, and *ABCG20* (Cohen et al. 2020). Suberin plays a vital role in forming a selective barrier for water and nutrient uptake while preventing excessive water loss and enhancing resistance to abiotic stresses (Chen, Liu, et al. 2022). These structural and molecular modifications may represent a key mechanism by which this type of plant extract strengthens the adaptive capacity of seedlings to adverse conditions, promoting more efficient and sustainable development.

Water relations in treated seedlings were markedly improved, as indicated by increased relative water content (RWC) and reduced osmotic potential (Ψs) (Figure 6A,B). These changes suggest enhanced water retention and improved osmotic adjustment. Up‐regulation of aquaporin genes in roots further underscores the effect of the extract facilitating water transport (Figure 6C). All *PIP aquaporins*, which are the main regulators of water movement for maintaining homeostasis in broccoli (Martinez‐Ballesta and Carvajal 2014), were *up‐regulated*. Additionally, all aquaporins that are basally expressed in roots (Nicolas‐Espinosa and Carvajal 2022) also showed increased expression, including *PIP1‐1b*, *PIP1‐2a*, *PIP1‐4*, *TIP2‐1b*, *TIP2‐3a*, *TIP4‐1a*, and *NIP5‐1a*. These findings underline the pivotal function of aquaporins in the development of the root system at approximately 10 days after germination, a stage where rapid cell division and elongation occur. Recent studies have revealed that aquaporins are involved not only in plant water relations but also in growth processes and development, such as tissue expansion (Maurel et al. 2008). The enhanced expression of both PIP and TIP aquaporins suggests a coordinated mechanism to optimize water uptake and intracellular water distribution, essential to meet the metabolic and structural demands of rapidly growing root tissue. This regulation likely not only improves water relations but also facilitates the uptake of nutrients, as aquaporins play an indirect but significant role in ensuring the transport of mineral solutes (Maurel et al. 2008). Such enhanced nutrient absorption is essential for sustaining the observed improvements in seedling growth and vigor under treatment.

In this regard, treated plants exhibited significant changes in mineral profiles. Macronutrients such as K, Ca, and Mg increased in both aerial and root tissues, while the Na/K ratio decreased, highlighting improved ion homeostasis and potential salt stress tolerance (Carillo et al. 2011). Micronutrient concentrations, including Cu, Fe, Mn, and Zn, also increased in aerial parts, supporting enhanced metabolic activities. These micronutrients play essential roles as cofactors for antioxidant enzymes: Cu, Mn, and Zn are integral to the activity of superoxide dismutase, while Fe and Cu contribute to the functions of catalase and peroxidase (Hänsch and Mendel 2009). This enzymatic activity reduces oxidative stress and may explain the reduction in lipid peroxidation previously reported (Liu et al. 2009).

In the kinetic essay, the glucosinolate content in the agar medium and seedlings decreased between days 3 and 7 (Figure 9), corresponding to their possible uptake and metabolization. This transient reduction was associated with the down‐regulation of sulfur assimilation genes like *APR2* and *APR3*, as well as key genes involved in feedback regulation, including *APK1* and *APK2* (Frerigmann 2016). The absence of these key isoforms, *APK1* and *APK2*, in *A. thaliana* , which are crucial for glucosinolate biosynthesis, has been shown to result in a general reduction of glucosinolate levels to approximately 20% of those in wild‐type plants. In addition, these mutants exhibited increased transcript levels of biosynthesis‐related genes (Mugford et al. 2009), which aligns with our RNAseq data (at day 10), showing reactivation of these genes, particularly those involved in aliphatic glucosinolate biosynthesis (Figure 8). This compensatory feedback mechanism suggests that the extract plays a dual role. Initially, it suppresses endogenous glucosinolate synthesis, possibly as an energy‐saving strategy (Bekaert et al. 2012; Popko et al. 2018), followed by a compensatory response triggered by the decline in glucosinolate levels.

**FIGURE 11:**
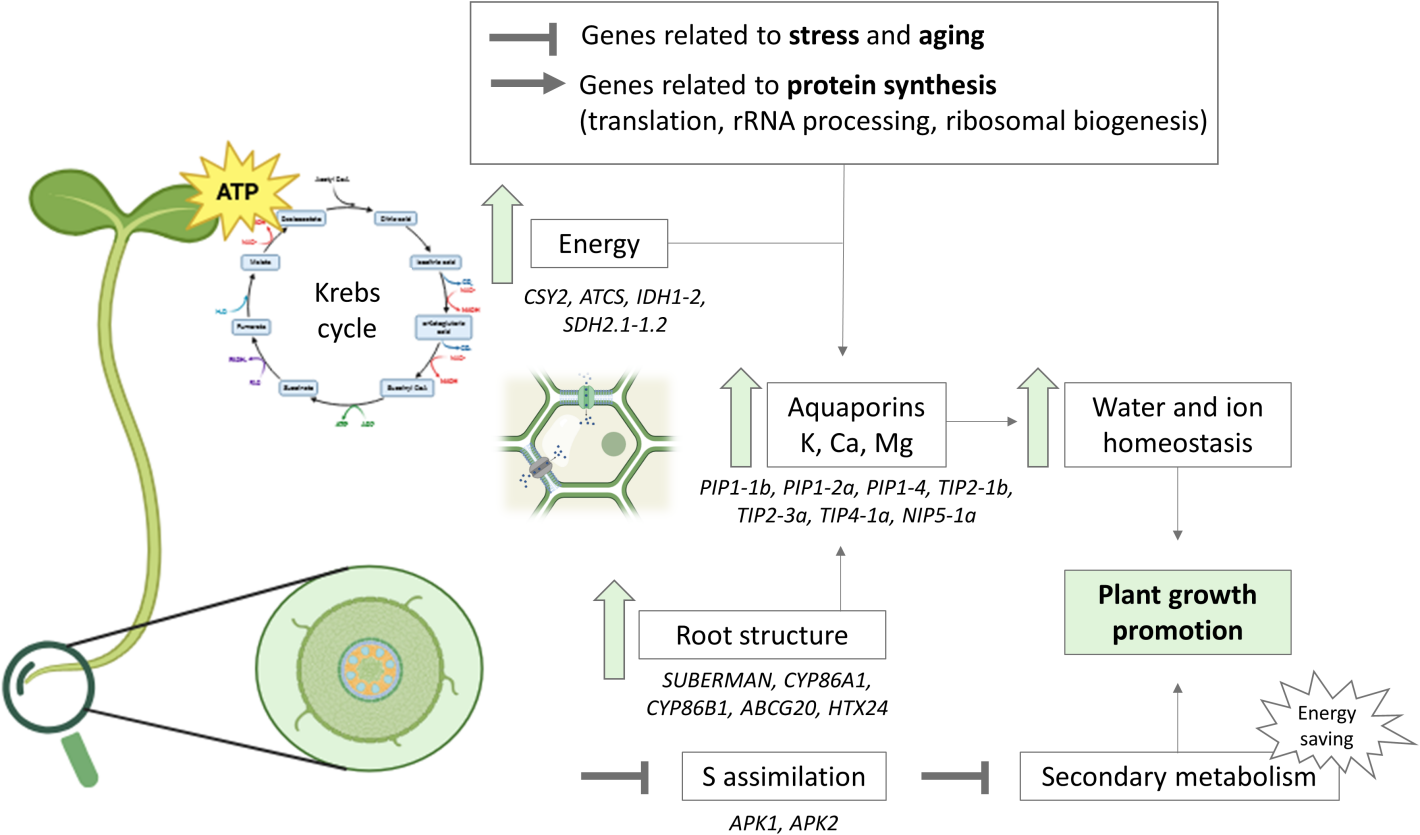
*Proposed mechanism of action* of broccoli extract rich in secondary metabolites in promoting seedling development.

This study demonstrates that the application of a glucosinolate‐rich plant extract promotes growth in broccoli seedlings by redirecting energy from stress responses to active development (Figure 11). The suppression of stress‐related and aging genes in both aerial and root tissues supports this shift from stress responses to active growth, further reinforced by the up‐regulation of genes involved in translation, rRNA processing, and ribosome biogenesis, indicating increased protein synthesis and cellular metabolic activity.

Enhanced metabolic efficiency was driven by up‐regulation of key Krebs cycle genes (*CSY2*, *ATCS*, *IDH1‐2*, *SDH2.1‐1.2*), supporting increased energy production. Root structure improvement, including enhanced suberin biosynthesis, was associated with the activation of genes such as *SUBERMAN* (*MYB39*), *CYP86A1*, *CYP86B1*, *ABCG20*, and *XTH24*, improving nutrient and water transport, which is also related to the up‐regulation of aquaporin genes. These molecular changes facilitated efficient water movement through the root system, supporting cellular hydration and the metabolic demands of rapidly growing seedlings. Additionally, the extract modulated glucosinolate metabolism, with suppression of sulfur assimilation genes (*APK1, APK2*) and up‐regulation of synthesis‐related genes, ensuring a balance between growth and physiological homeostasis of broccoli seedlings.

Overall, these findings provide valuable insights into the molecular mechanisms underlying biostimulant activity in plants, highlighting the potential of glucosinolate‐rich extracts as sustainable tools to enhance crop performance. Future studies should explore the long‐term effects of such extracts under field conditions and evaluate their efficacy across different plant species and environmental contexts to support their broader application in sustainable agriculture.

## Author Contributions

**Lorena Albaladejo‐Marico:** formal analysis, investigation, writing – original draft. **Micaela Carvajal:** conceptualization, validation, writing – review and editing, project administration, supervision, funding acquisition. **Lucia Yepes‐Molina:** conceptualization, methodology, validation, formal analysis, writing – review and editing, supervision.

## Supporting information


**Figure S1.** Gene ratio plot illustrating the biological process (BP) terms in the aerial part (A) and root (B) of extract‐treated seedlings compared to control plants. The y‐axis shows the names of the KEGG terms, while the x‐axis represents the gene ratio. The size of the circle corresponds to the gene count, and the intensity of the colour indicates the adjusted *p* value.
**Figure S2.** Heatmap of aquaporins gene expression in extract‐treated and control seedlings in the aerial part. Blue represents genes with low expression and red represents genes with high expression (*Z*‐score); fold change (logFC), mean expression (AveExpr) and gene name are shown. Significant differences between extract‐treated and control seedlings were measured by *t*‐tests. *p < 0.05, **p < 0.01, ***p < 0.001.
**Figure S3.** Mineral concentrations in the aerial and root part of the seedlings treated with extract compared to the control. Each value represents the mean ± SE (*n* = 3). Significant differences between extract‐treated and control plants were measured by *t*‐tests. *p < 0.05, ** *p* < 0.01, ***p < 0.001.
**Figure S4.** Heatmap of mineral transporter gene expression in extract‐treated and control seedlings in (A) the aerial part and (B) root. Blue represents genes with low expression and red represents genes with high expression (*Z*‐score); fold change (logFC), mean expression (AveExpr) and gene name are shown. Significant differences between extract‐treated and control plants were measured by *t*‐tests. *p < 0.05, **p < 0.01, ***p < 0.001.
**Figure S5.** Heatmap of gene expression related to glucosinolate synthesis and regulation in extract‐treated and control seedlings in the aerial part (A) and root (B). Heat map of gene expression related to phenol synthesis and regulation in extract‐treated and control seedlings in the aerial part (C) and root (D). Blue represents genes with low expression and red represents genes with high expression (*Z*‐score); fold change (logFC), mean expression (AveExpr) and gene name are shown. Significant differences between extract treated and control plants were measured by *t*‐tests. *p < 0.05, **p < 0.01, ***p < 0.001.
**Figure S6.** Heatmap of gene expression related to auxin synthesis and regulation in extract‐treated and control seedlings in (A) the aerial part and (B) root. Summary table (GO terms) of the functions of the auxin genes studied. Heat map of gene expression related to gibberellins and zeatin synthesis and regulation in extract‐treated and control seedlings in (C) the aerial part and (D) root. Blue represents genes with low expression and red represents genes with high expression (*Z*‐score); fold change (logFC), mean expression (AveExpr) and gene name are shown. Significant differences between extract treated and control plants were measured by t‐tests. *p < 0.05, **p < 0.01, ***p < 0.001.
**Table S1.** Summary of the sequencing data generated for RNA‐seq and mapping of the Broccoli genome.

## Data Availability

The omics data that support the findings of this study are openly available in NCBI at reference number PRJNA1231065. The other data that support the findings of this study are available from the corresponding author upon reasonable request.
