# Supplementary material for: Bioactive Glucosinolate‐Rich Extract Promotes Growth in Broccoli Seedlings by Modulating Energy Allocation
Source: Physiol Plant. 2025 Jul 12;177(4):e70391. doi: 10.1111/ppl.70391 (PMC12254940; doi:10.1111/ppl.70391)
Supplement: Supplementary file 1 — Figure S1. Gene ratio plot illustrating the biological process (BP) terms in the aerial part (A) and root (B) of extract‐treated seedlings compared to control plants. The y‐axis shows the names of the KEGG terms, while the x‐axis represents the gene ratio. The size of the circle corresponds to the gene count, and the intensity of the colour indicates the adjusted p value. Figure S2. Heatmap of aquaporins gene expression in extract‐treated and control seedlings in the aerial part. Blue represents genes with low expression and red represents genes with high expression (Z‐score); fold change (logFC), mean expression (AveExpr) and gene name are shown. Significant differences between extract‐treated and control seedlings were measured by t‐tests. *p < 0.05, **p < 0.01, ***p < 0.001. Figure S3. Mineral concentrations in the aerial and root part of the seedlings treated with extract compared to the control. Each value represents the mean ± SE (n = 3). Significant differences between extract‐treated and control plants were measured by t‐tests. *p < 0.05, ** p < 0.01, ***p < 0.001. Figure S4. Heatmap of mineral transporter gene expression in extract‐treated and control seedlings in (A) the aerial part and (B) root. Blue represents genes with low expression and red represents genes with high expression (Z‐score); fold change (logFC), mean expression (AveExpr) and gene name are shown. Significant differences between extract‐treated and control plants were measured by t‐tests. *p < 0.05, **p < 0.01, ***p < 0.001. Figure S5. Heatmap of gene expression related to glucosinolate synthesis and regulation in extract‐treated and control seedlings in the aerial part (A) and root (B). Heat map of gene expression related to phenol synthesis and regulation in extract‐treated and control seedlings in the aerial part (C) and root (D). Blue represents genes with low expression and red represents genes with high expression (Z‐score); fold change (logFC), mean expression (AveExpr) and gene nam [file PPL-177-e70391-s001.pdf]

Bioactive glucosinolate-rich extract promotes growth in broccoli seedlings by modulating energy allocation

Lorena Albaladejo-Marico, Micaela Carvajal, Lucia Yepes-Molina *

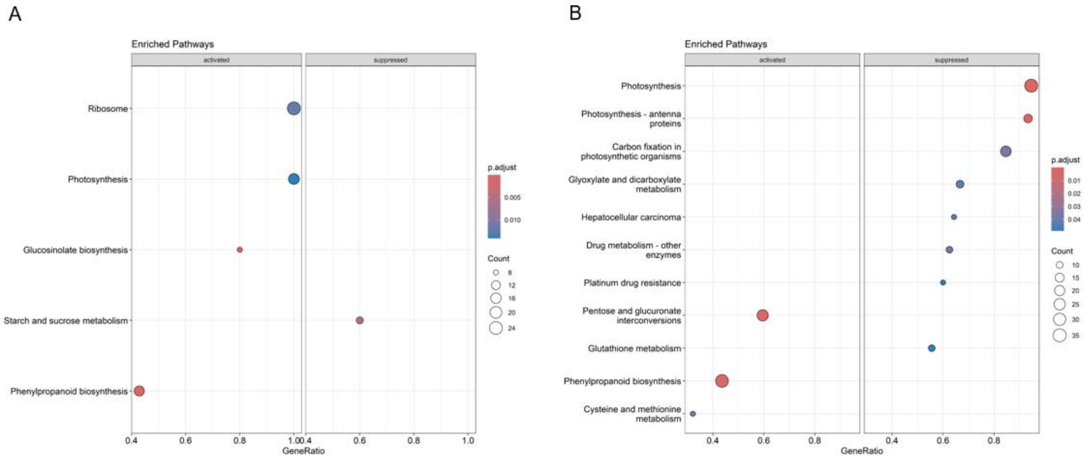

***Figure S1**. Gene ratio plot illustrating the biological process (BP) terms in the aerial part **(A)** and root **(B)** of extract-treated seedlings compared to control plants. The y-axis shows the names of the KEGG terms, while the x-axis represents the gene ratio. The size of the circle corresponds to the gene count, and the intensity of the colour indicates the adjusted p-value.*

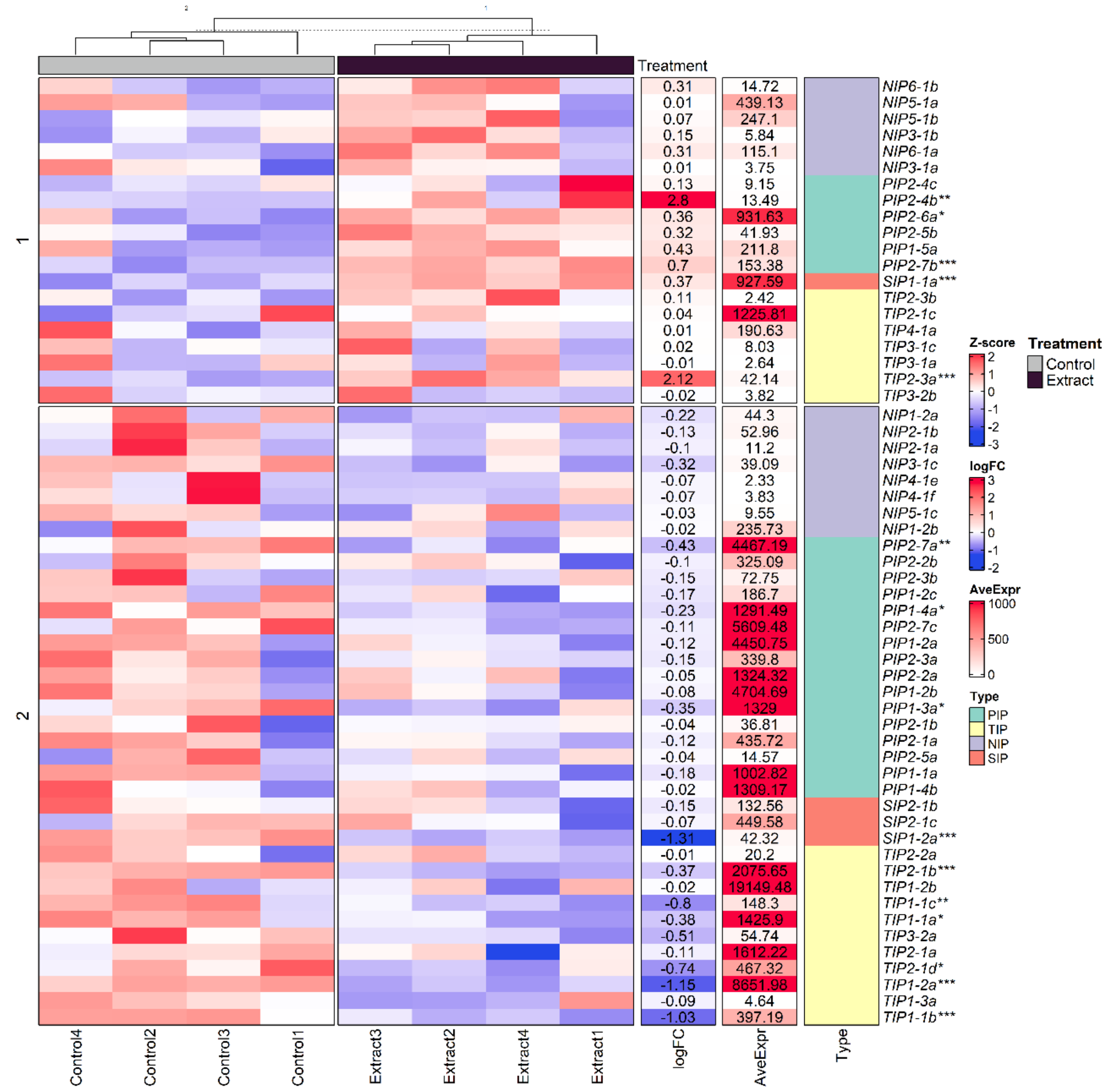

Figure S2. *Heatmap of aquaporins gene expression in extract-treated and control seedlings in the aerial part. Blue represents genes with low expression and red represents genes with high expression (Z-score); fold change (logFC), mean expression (AveExpr) and gene name are shown. Significant differences between extract-treated and control seedlings were measured by t-tests. * $p < 0.05$, ** $p < 0.01$, *** $p < 0.001$.*

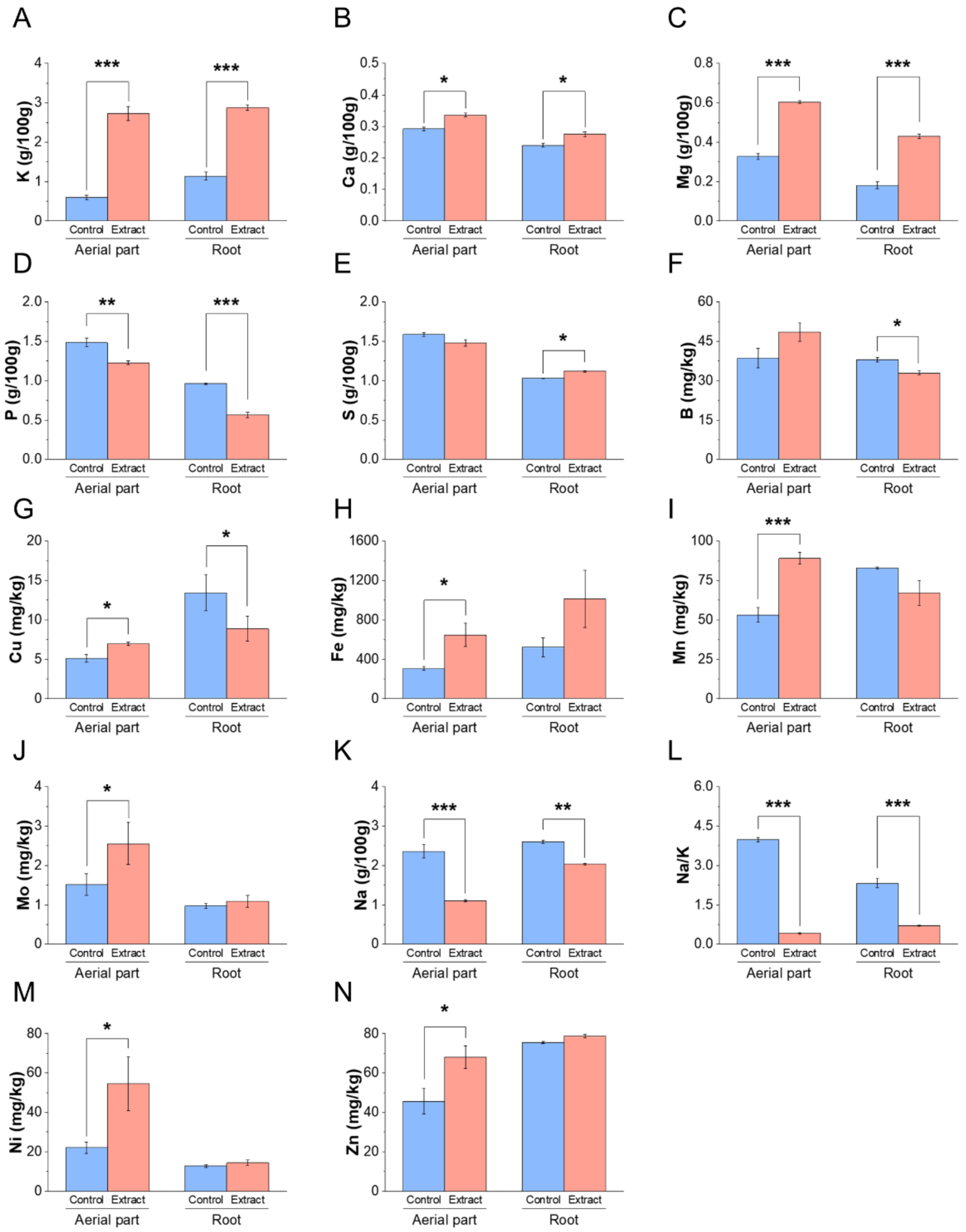

Figure S3. *Mineral concentrations in the aerial and root part of the seedlings treated with extract compared to the control. Each value represents the mean ± SE (n = 3).Significant differences between extract-treated and control plants were measured by t-tests. * p < 0.05, ** p < 0.01, *** p < 0.001.*

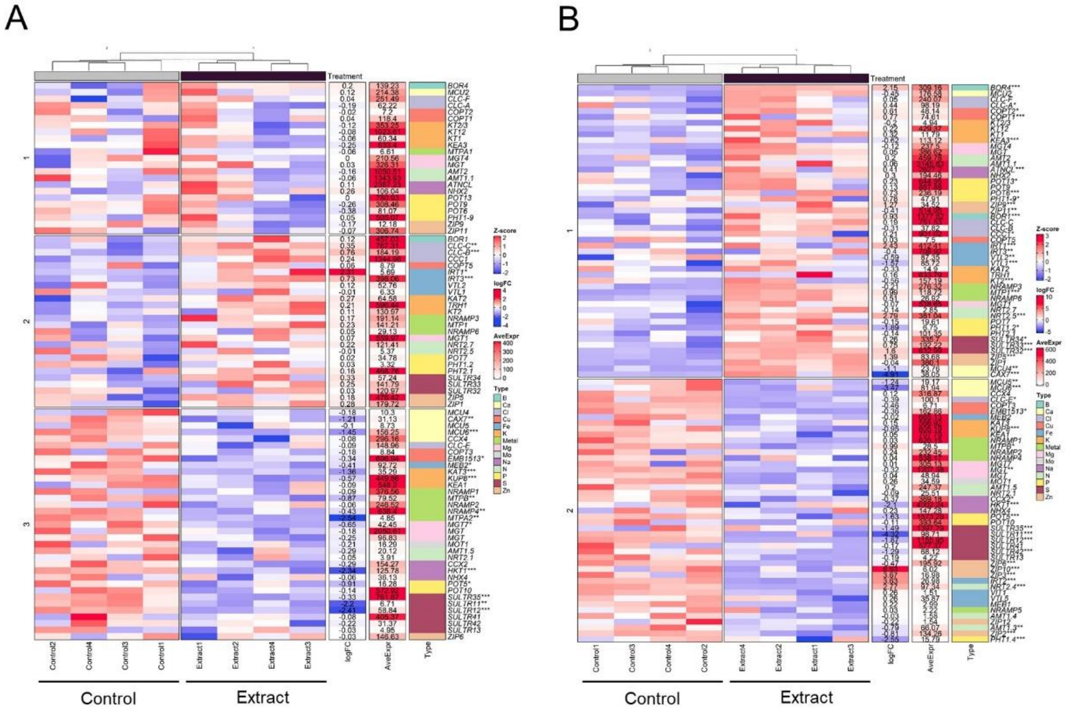

Figure S4. *Heatmap of mineral transporter gene expression in extract-treated and control seedlings in* ***A)*** *the aerial part and* ***B)*** *root. Blue represents genes with low expression and red represents genes with high expression (Z-score); fold change (logFC), mean expression (AveExpr) and gene name are shown. Significant differences between extract-treated and control plants were measured by t-tests. * $p < 0.05$, ** $p < 0.01$, *** $p < 0.001$.*

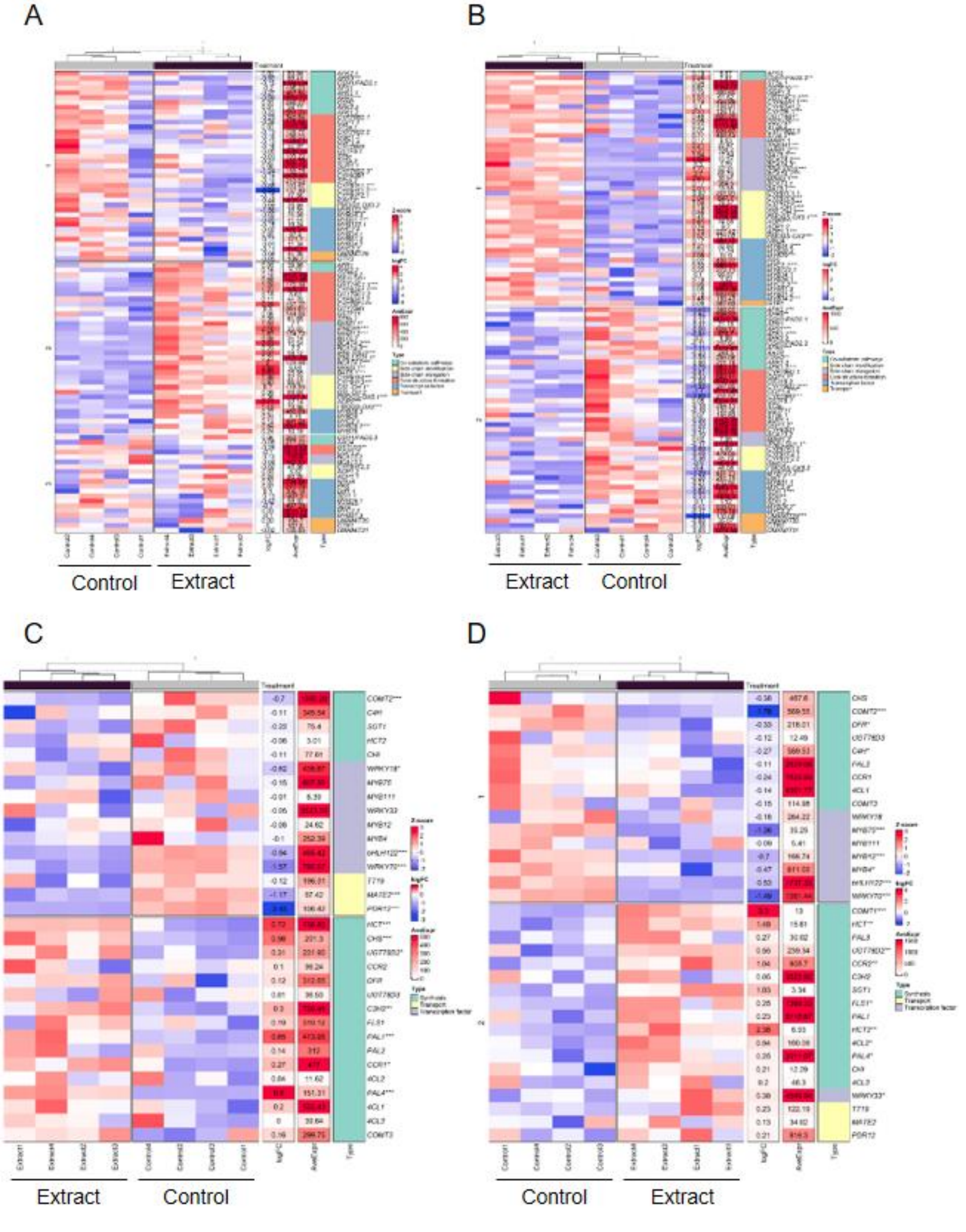

Figure S5*. Heatmap of gene expression related to glucosinolate synthesis and regulation in extract-treated and control seedlings in the aerial part* ***(A)*** *and root* ***(B)****. Heat map of gene expression related to phenol synthesis and regulation in extract-treated and control seedlings in the aerial part* ***(C)*** *and root* ***(D)****. Blue represents genes with low expression and red represents genes with high expression (Z-score); fold change (logFC), mean expression (AveExpr) and gene name are shown. Significant differences between extract treated and control plants were measured by t-tests. * $p < 0.05$, ** $p < 0.01$, *** $p < 0.001$.*

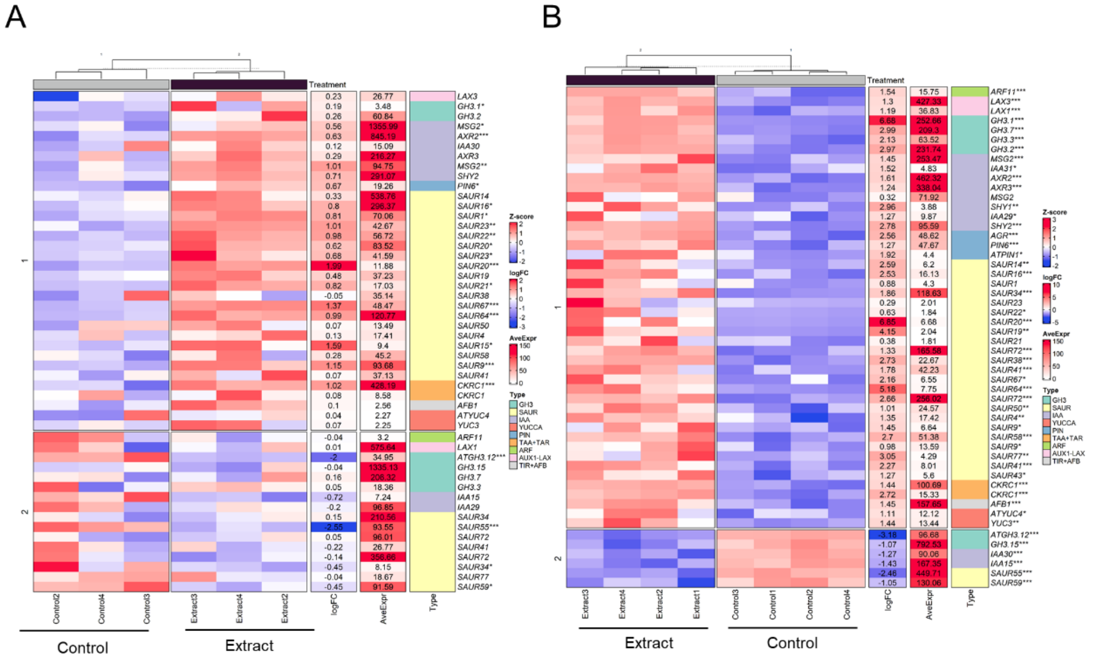

ID	Description	p. value
GO:0009630	Gravitropism	2.97E-19
GO:0009606	Tropism	1.72E-18
GO:0009629	Response to gravity	2.34E-18
GO:0060918	Auxin transport	2.65E-14
GO:0009914	Hormone transport	4.30E-14

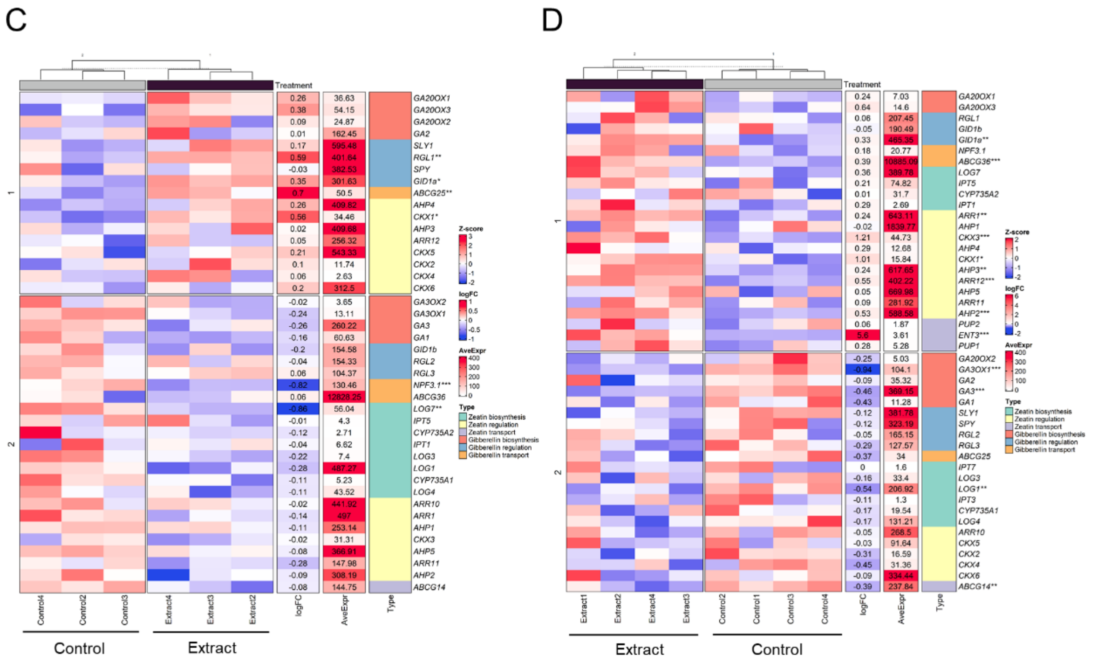

Figure S6*. Heatmap of gene expression related to auxin synthesis and regulation in extract-treated and control seedlings in* ***A)*** *the aerial part and* ***B)*** *root. Summary table (GO terms) of the functions of the auxin genes studied. Heat map of gene expression related to gibberellins and zeatin synthesis and regulation in extract-treated and control seedlings in* ***C)*** *the aerial part and* ***D)*** *root. Blue represents genes with low expression and red represents genes with high expression (Z-score); fold change (logFC), mean expression (AveExpr) and gene name are shown. Significant differences between extract treated and control plants were measured by t-tests. * $p < 0.05$, ** $p < 0.01$, *** $p < 0.001$.*

__Table S1.__ Summary of the sequencing data generated for RNA-seq and mapping of the Broccoli genome.

Sample	Rep	Tissue	Raw Reads	Clean Reads	GC Content (%)	% ≥ Q30	Mapped Reads (%)
**C2-R**	1	root	34386698	33604788	46.78	91.09	94.44
**C3-R**	2	root	41600372	39813632	46.38	90.61	93.39
**C5-R**	3	root	60726014	52449578	46.06	88.11	87.61
**C4-R**	4	root	43334874	38720562	46.84	88.14	94.5
**C4-A**	1	aerial-part	52682438	52520222	47.59	88.32	89.55
**C5-A**	2	aerial-part	63446996	55994562	47.07	87.89	89.16
**C1-A**	3	aerial-part	49725700	45517894	46.51	88.97	90.66
**C3-A**	4	aerial-part	55200000	47974768	47.06	88.06	88.65
**E3-R**	1	root	44594912	40294746	46.95	87.78	90.21
**E5-R**	2	root	50838062	45357952	46.11	88.19	89.04
**E4-R**	3	root	49114604	43257928	46.19	87.95	88.57
**E6-R**	4	root	43602868	43261992	47.36	87.46	85.97
**E2-A**	1	aerial-part	50049562	44693354	46.74	88.30	89.22
**E1-A**	2	aerial-part	51884780	46382450	46.92	88.30	89.27
**E4-A**	3	aerial-part	54934906	48757166	46.96	88.16	89.11
**E5-A**	4	aerial-part	46656570	42003960	46.88	89.10	90.59
